# Biomimetic Zirconia with Cactus-Inspired Meso-Scale Spikes and Nano-Trabeculae for Enhanced Bone Integration

**DOI:** 10.3390/ijms22157969

**Published:** 2021-07-26

**Authors:** Juri Saruta, Ryotaro Ozawa, Takahisa Okubo, Samira R. Taleghani, Manabu Ishijima, Hiroaki Kitajima, Makoto Hirota, Takahiro Ogawa

**Affiliations:** 1 Weintraub Center for Reconstructive Biotechnology, Division of Advanced Prosthodontics, UCLA School of Dentistry, Los Angeles, CA 90095-1668, USA; saruta@kdu.ac.jp (J.S.); ozrt1021@gmail.com (R.O.); okubotakahisa@gmail.com (T.O.); s.r.taleghani@gmail.com (S.R.T.); manab612@gmail.com (M.I.); hiroaki_k_0315@yahoo.co.jp (H.K.); mhirota@yokohama-cu.ac.jp (M.H.); 2 Department of Education Planning, Kanagawa Dental University, 82 Inaoka, Yokosuka 238-8580, Kanagawa, Japan; 3 Department of Oral Interdisciplinary Medicine (Prosthodontics & Oral Implantology), Graduate School of Dentistry, Kanagawa Dental University, 82 Inaoka, Yokosuka 238-8580, Kanagawa, Japan; 4 Department of Partial Denture Prosthodontics, Nihon University School of Dentistry, 1-8-13 Kanda Surugadai, Chiyoda-ku, Tokyo 101-8310, Japan; 5 Department of Oral and Maxillofacial Surgery, Graduate School of Medicine, Yokohama City University, 3-9 Fukuura, Kanazawa-ku, Yokohama 236-0004, Kanagawa, Japan; 6 Department of Oral and Maxillofacial Surgery/Orthodontics, Yokohama City University Medical Center, 4-57 Urafune-cho, Minami-ku, Yokohama 232-0024, Kanagawa, Japan

**Keywords:** bone-implant integration, Y-TZP, dental and orthopedic implant, laser etching

## Abstract

Biomimetic design provides novel opportunities for enhancing and functionalizing biomaterials. Here we created a zirconia surface with cactus-inspired meso-scale spikes and bone-inspired nano-scale trabecular architecture and examined its biological activity in bone generation and integration. Crisscrossing laser etching successfully engraved 60 μm wide, cactus-inspired spikes on yttria-stabilized tetragonal zirconia polycrystal (Y-TZP) with 200–300 nm trabecular bone-inspired interwoven structures on the entire surface. The height of the spikes was varied from 20 to 80 μm for optimization. Average roughness (Sa) increased from 0.10 μm (polished smooth surface) to 18.14 μm (80 μm-high spikes), while the surface area increased by up to 4.43 times. The measured dimensions of the spikes almost perfectly correlated with their estimated dimensions (R^2^ = 0.998). The dimensional error of forming the architecture was 1% as a coefficient of variation. Bone marrow-derived osteoblasts were cultured on a polished surface and on meso- and nano-scale hybrid textured surfaces with different spike heights. The osteoblastic differentiation was significantly promoted on the hybrid-textured surfaces compared with the polished surface, and among them the hybrid-textured surface with 40 μm-high spikes showed unparalleled performance. In vivo bone-implant integration also peaked when the hybrid-textured surface had 40 μm-high spikes. The relationships between the spike height and measures of osteoblast differentiation and the strength of bone and implant integration were non-linear. The controllable creation of meso- and nano-scale hybrid biomimetic surfaces established in this study may provide a novel technological platform and design strategy for future development of biomaterial surfaces to improve bone integration and regeneration.

## 1. Introduction

Bio-inspired or biomimetic design of biomaterials presents new possibilities for developing implantable devices with enhanced biocompatibility and novel functions [1,2,3,4,5,6,7,8]. No study has yet reported a potential application of biomimetic surface morphology, particularly at the nano-level, to endosseous implants for commercial use in the fields of dental and orthopedic surgery [9,10,11,12,13,14,15,16,17,18,19,20]. Most of the advancements thus far in implant surface design to improve their ability to integrate with bone can be characterized as development of micro-topography to enhance osteoblastic function [21,22,23,24,25,26,27,28,29,30,31,32]. Specifically, micro-scale roughness, ranging from 0.5 μm to 5 μm, made by various chemical and physical treatments has been shown to successfully promote osteoblastic differentiation, thereby leading to faster and firmer bone integration than a machined-smooth surface [22,23,25,28,30,31]. A major challenge remains unsolved with respect to how distinct meso- (10 to 500 μm) and nano-scale surface topography can be created, and more importantly, the osteoblastic reaction to these scales of morphology/roughness is largely untested. Establishing a technological platform and accompanying design strategy on an experimental yet scalable manufacturing level would provide an initial solution for these important outstanding questions in the field.

A recent laser technology advancement made a breakthrough in simultaneous creation of meso- and nano-scale topography on zirconia [20]. Zirconia, made with yttria-stabilized tetragonal zirconia polycrystal (Y-TZP), is an allergy-free implantable material which is biocompatible with bone [20,33]. Optimizing conditions for the solid-state laser etching has enabled the engraving of meso-scale grooves with unique nanostructures upraised from their surfaces [20]. It was demonstrated that the width and depth of these meso-scale grooves can be controlled. The next step is to harness the technology for a novel surface design to further improve cellular and tissue reaction. For application to dental and orthopedic implants, a specific goal is to enhance bone–implant integration. A possible strategy from a mechanical perspective is to effectively increase the surface area of the implant and also the mechanical interlocking between the implant and bone, and we hypothesized that meso-scale texture holds a key to accomplishing this strategy. From a biological perspective, we hypothesized that the simultaneous presence of optimized meso-scale and nano-scale textures would promote osteoblastic differentiation, and furthermore, that if the nano-texture could create a bio-inspired local environment, it would be even more effective. In pursuit of these goals, we created a novel zirconia surface with cactus-like meso-scale spikes and a distinct nano-scale topography of trabecular bone-like morphology by solid-state laser etching. We then optimized the meso- and nano-scale hybrid textured surface for better bone integration. Further, we validated the technology by developing a prototype dental implant with the bio-inspired optimized hybrid texture.

## 2. Results

### 2.1. Creation of Bio-Inspired Meso-/Nano-Scale Hybrid Textured Zirconia

**Figure 1:**
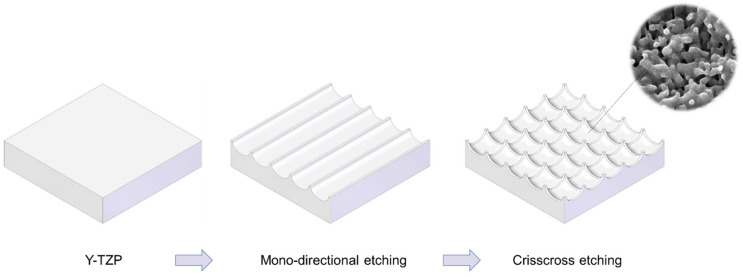
Schematic illustration of design strategy used to create cactus-inspired meso-scale spikes. Crisscrossing lattice grooves are engraved by vertical and horizontal laser etching. The grooves are hemispherical, leaving unengraved areas projecting as spikes. The width and depth of the grooves are controllable; the width was fixed at 60 μm in the present study, while the depth was varied in 5 increments from 20 to 80 μm.

**Figure 2:**
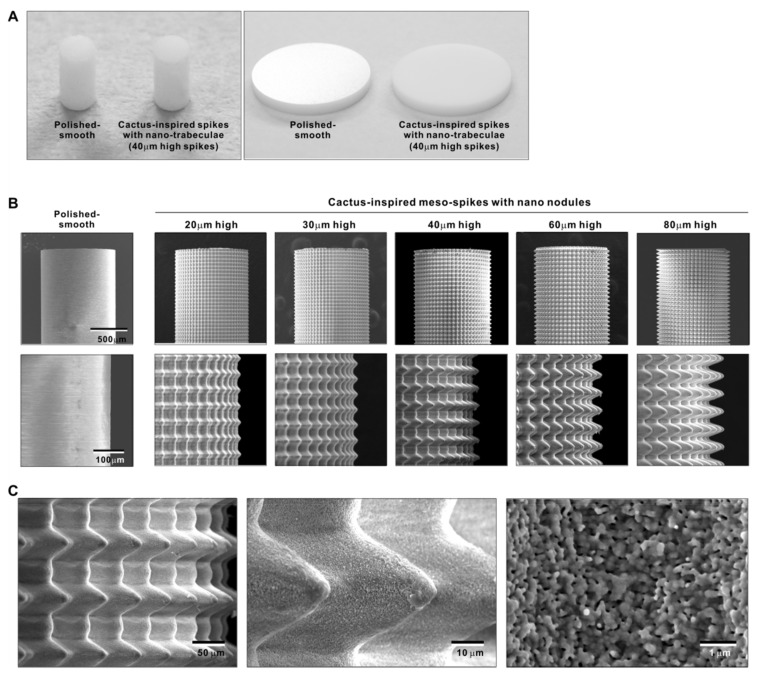
Creation of bio-inspired meso- and nano-scale hybrid textured zirconia. (**A**) Photographs of experimental zirconia samples made in cylinder and disk forms. Shown are a polished-smooth surface and a 40 μm-high spiked surface as a representative laser-etched zirconia sample. (**B**) Low-magnification SEM images of the polished cylinder zirconia and the meso- and nano-scale textured cylindrical zirconia with various heights of meso-spikes. The meso-scale spikes resembled cactus prickles. (**C**) High-magnification SEM images of the meso- and nano-scale textured zirconia with 40 μm-high spikes, vividly capturing the co-existence of cactus-inspired meso-spikes and nano-scale interwoven architecture resembling trabecular bone.

We attempted to create zirconia surfaces with cactus-like meso-scale spikes and nano-scale architecture using crisscrossing solid-state laser etching, as illustrated in Figure 1. The meso-scale spikes were designed with 60 μm width and five different heights of 20, 30, 40, 60, and 80 μm. Zirconia experimental samples in cylindrical and disk forms were made from yttria-stabilized tetragonal zirconia polycrystal (Y-TZP) (Figure 2A). The zirconia samples roughened through laser etching appeared to reflect less light than polished-smooth zirconia. Figure 2B (top images) shows low-magnification SEM images of zirconia cylinders with different meso-spike heights created by laser etching as well as the polished-smooth zirconia without laser etching. Laser-etched zirconia samples exhibited uniform, even, and seamless formation of spikes all over their circumference, regardless of the spike height. High-magnification images of these zirconia cylinders vividly revealed the formation of cactus-like spikes with consistent size and height, and in perfect lattice alignment (bottom images in Figure 2B). By controlling laser conditions, meso-scale spikes of gradually increased height were successfully created, while the width was fixed to approximately 60 μm. Even higher-magnification images revealed the formation of randomly shaped, densely networked nanostructures all over the surfaces of spikes of all heights; the nano-architecture resembled trabecular bone (Figure 2C). The trabeculae-like structures were 100 to 400 nm in size and appeared assembled and upraised from the base zirconia.

### 2.2. Quantitative Assessment of Surface Roughness of Bio-Inspired Meso-/Nano-Scale Hybrid Textured Zirconia

**Figure 3:**
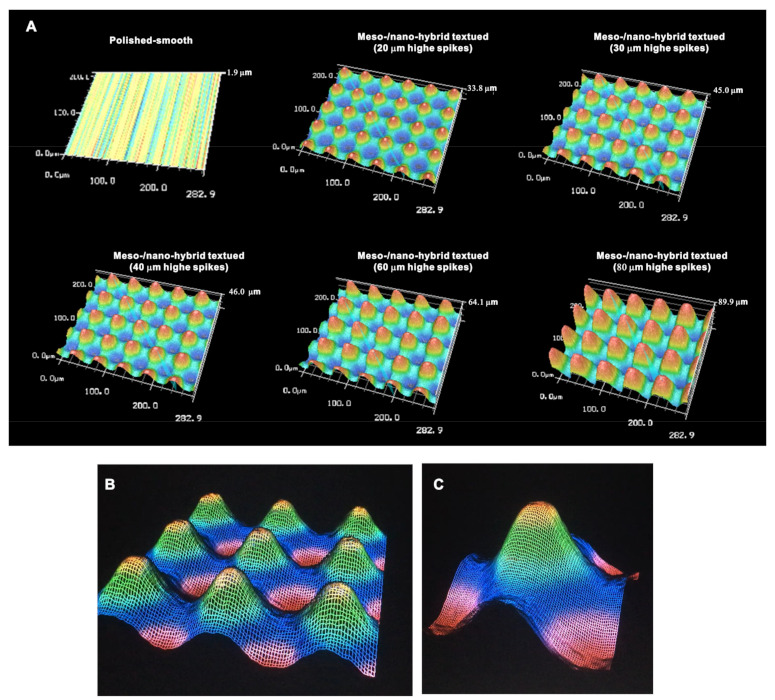
Three-dimensional profiles of the bio-inspired meso- and nano-scale hybrid textured zirconia. (**A**) Three-dimensional images of the polished zirconia and the meso- and nano-scale textured zirconia with various heights of meso-spikes. (**B**,**C**) Close-up images of the 40 μm-high meso-spikes, depicting precise and smooth transitions from peaks to valleys.

We continued analyzing the meso-/nano-scale hybrid textured zirconia. Three-dimensional images captured 3-D projection of the meso-scale spikes with heights incrementally increased from 20 to 80 μm (Figure 3A). Zoomed-in views of the meso-spiked surfaces showed that the laser etching produced highly controlled, smooth outlines oscillating from peaks to valleys (Figure 3B,C).

**Figure 4:**
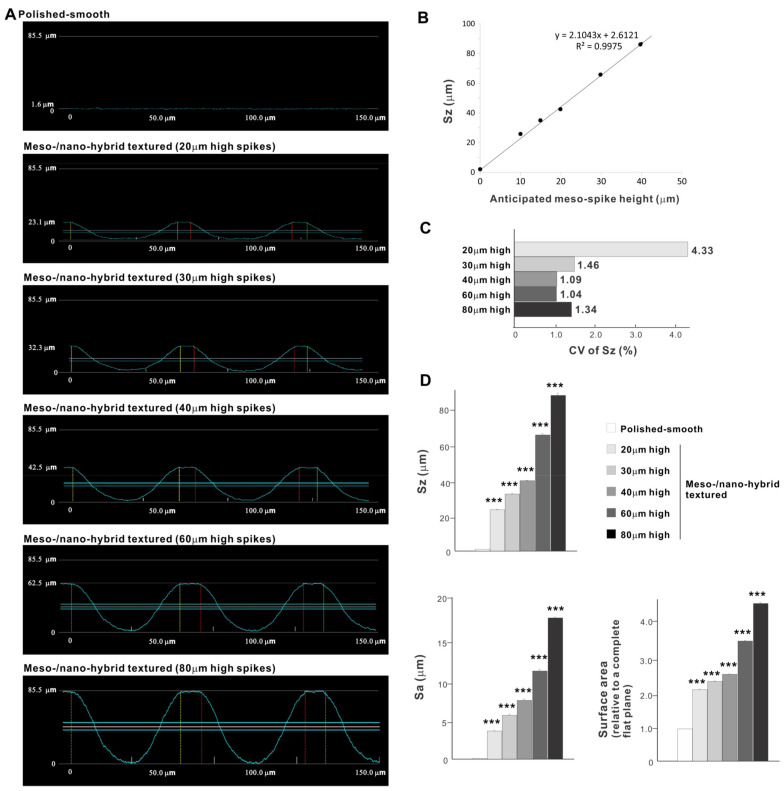
Quantitative surface analyses of the bio-inspired meso-/nano-scale hybrid textured zirconia. (**A**) Cross-sectional profile curves of the polished-smooth zirconia and the meso-/nano-scale textured zirconia with various heights of meso-spikes. (**B**) Sz (peak-to-valley roughness) plotted against the estimated/planned height of meso-scale spikes, showing a near-perfect linear correlation. (**C**) Coefficient of variation (CV) of Sz for different height designs of meso-scale spikes. CV (%) = (SD/Mean) × 100. (**D**) Quantitative topographical evaluations; Sz, Sa (average roughness), and surface area. *** *p* < 0.001, statistically significant difference compared with a polished surface.

Cross-sectional profiling along the line connecting spike peaks confirmed smooth, oscillating curves of spikes and valleys (Figure 4A). The width of a spike was stable at approximately 60 μm for all height variations. The heights of spikes measured by profiling and the average peak-to-valley roughness (Sz) nearly matched the anticipated heights, with the measured heights of 23.1, 32.3, 42.5, 62.5, and 85.5 μm for the anticipated heights of 20, 30, 40, 60, and 80 μm, respectively (Figure 4A,B). There was a very high correlation between the measured spike height (Sz) and the anticipated height (R^2^ = 0.9975; Figure 4B). The coefficient of variation for Sz was less than 1.5% for 30, 40, 60, and 80 μm-high spikes and near 1.0% for 40 and 60 μm-high spikes (Figure 4C). These data indicate the precision and reproducibility of the surface texturing process. Sz, average roughness (Sa), and surface area increased dramatically with the addition of meso-spikes and further increased in proportion to the incremental height increase of the meso-spikes (Figure 4D). Of significance, Sa exceeded 5.0 μm when the spike height was 30 μm or higher, followed by a further increase to over 15 μm. The surface areas of samples with 20, 30, and 40 μm-high spikes were more than twice that of the polished surface and increased to 4.43 times that of the polished surface with 80 μm-high spikes.

### 2.3. Osteoblast Attachment and Proliferation on Meso-/Nano-Scale Hybrid Textured Zirconia

**Figure 5:**
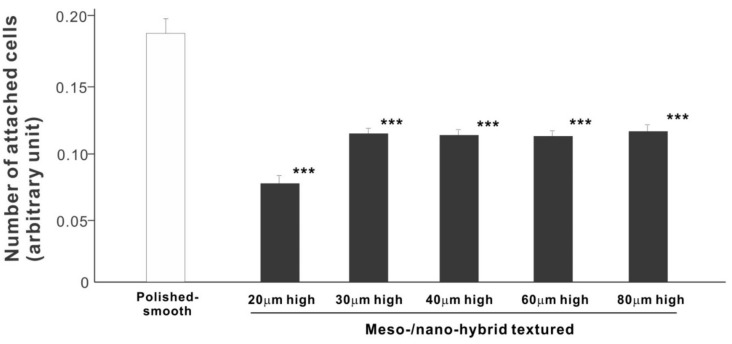
Attachment of osteoblasts to differently textured zirconia surfaces during the initial stage of culture, evaluated by WST-1 assay on day 2. *** *p* < 0.001, statistically significant difference compared with a polished surface.

In a quest to optimize a zirconia surface for the promotion of bone–implant integration, we cultured osteoblasts on meso-/nano-scale hybrid textured zirconia with various spike heights as well as on a polished-smooth zirconia surface. The number of osteoblasts attached to the zirconia surfaces was evaluated during the initial stage of culture on day 2. All hybrid-textured zirconia surfaces showed a significantly lower number of attached cells than on the polished surface (Figure 5), and among these, the surface with 20 μm-high spikes showed the lowest number of attached cells. There were no statistically significant differences among the rest of the hybrid-textured zirconia samples.

**Figure 6:**
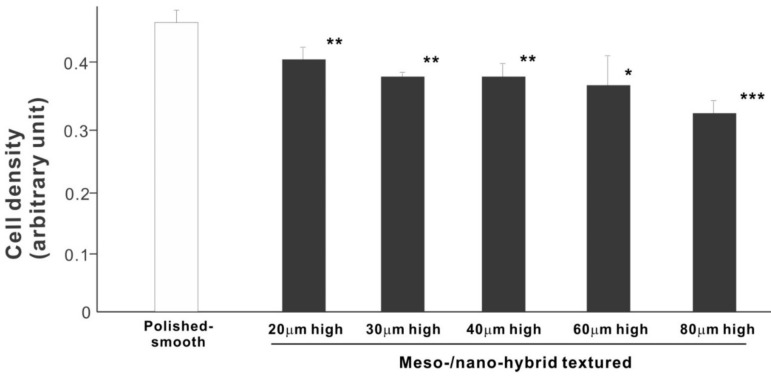
Proliferation of osteoblasts, measured as cell density, on differently textured zirconia surfaces, evaluated by the WST-1 assay on day 5. * *p* < 0.05, ** *p* < 0.01, *** *p* < 0.001, statistically significant difference compared with a polished surface.

We then examined the ability of the zirconia samples to support cell proliferation by measuring the density of cells on day 5 of culture (Figure 6). The cell density on all hybrid-textured zirconia samples was significantly lower than on the polished surface. The difference between the polished surface and hybrid surfaces was diminished compared to the initial cell attachment evaluated on day 2. A decreasing trend was found on the hybrid surfaces with higher spikes, although there was no significant difference among the surfaces with 20, 30, 40, and 60 μm-high spikes.

### 2.4. Osteoblast Differentiation on Meso-/Nano-Scale Hybrid Textured Zirconia

**Figure 7:**
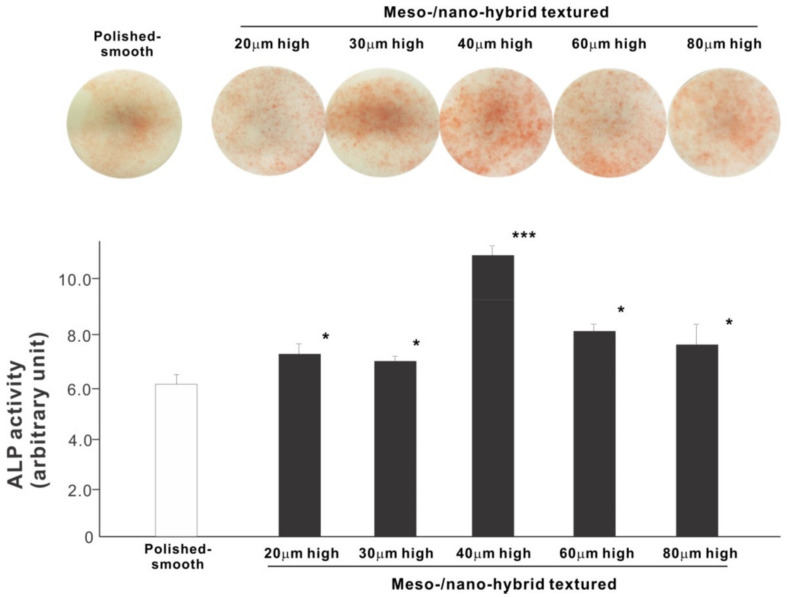
Osteoblastic differentiation on variously textured zirconia surfaces evaluated by alkaline phosphatase (ALP) activity on day 10 of culture. Culture images after ALP staining (top images) and the amount of ALP (histogram) measured by chemical detection are presented. * *p* < 0.05, *** *p* < 0.001, statistically significant difference compared with a polished surface.

**Figure 8:**
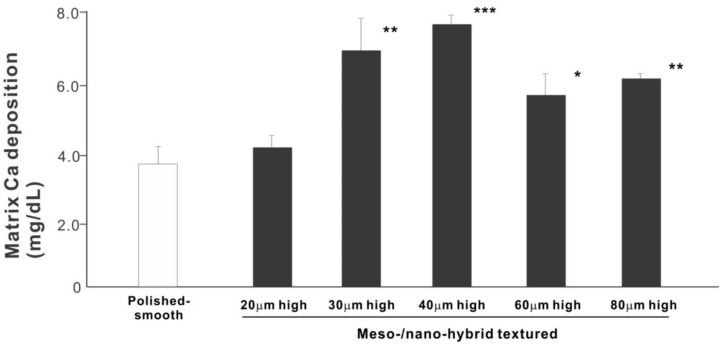
Osteoblastic differentiation on variously textured zirconia surfaces evaluated by matrix calcium deposition on day 15 of culture. * *p* < 0.05, ** *p* < 0.01, *** *p* < 0.001, statistically significant difference compared with a polished surface.

We next examined osteoblastic differentiation by measuring ALP activity, matrix mineralization ability, and the expression levels of osteoblastic differentiation marker genes. Unlike the results in the cell attachment and cell density assays, the ALP activity measured on day 10 was significantly higher for all meso-/nano-scale hybrid textured zirconia than on the polished surface (histogram in Figure 7). There was a disproportional change of ALP activity relative to the spike height, with the ALP activity being the highest when the spikes were 40 μm high. The result of ALP staining of the culture confirmed these results (top images in Figure 7). The matrix calcium deposition measured on day 20 also dominated on the hybrid surfaces and seemed to increase linearly with the spike height up to a certain point, followed by a decline (Figure 8). Specifically, the peak of the calcium deposition was found with 40 μm-high spikes.

**Figure 9:**
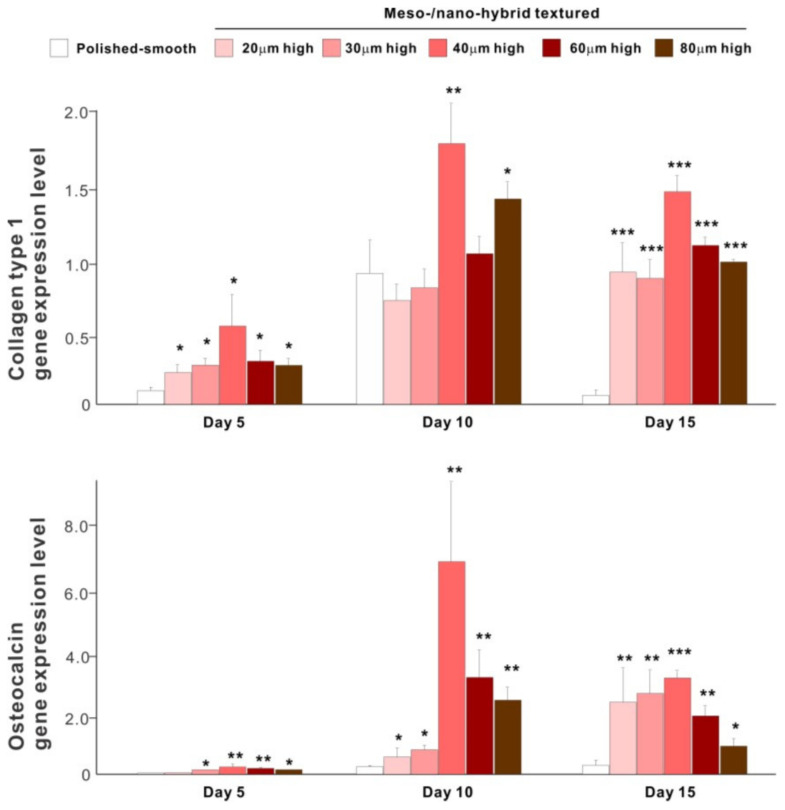
Osteoblastic differentiation on variously textured zirconia surfaces evaluated by real-time PCR on days 5, 10, and 15 of culture. Collagen type 1 and osteocalcin gene expression were evaluated as early- and late-stage differentiation markers, respectively. * *p* < 0.05, ** *p* < 0.01, *** *p* < 0.001, statistically significant difference compared with a polished surface.

The results of real-time PCR resembled those of the matrix calcium deposition (Figure 9). The expression of collagen type 1 gene, an early-stage osteoblastic marker, was generally upregulated on the hybrid surfaces compared with the polished surface throughout the culture period. The expression level increased with the height of meso-spikes and was the highest with 40 μm-high spikes at all time points tested. The expression of osteocalcin, a late-stage osteoblastic marker, was significantly higher on the hybrid surfaces and most upregulated on the 40 μm-high spiked surface.

### 2.5. In Vivo Bone-and-Implant Integration

**Figure 10:**
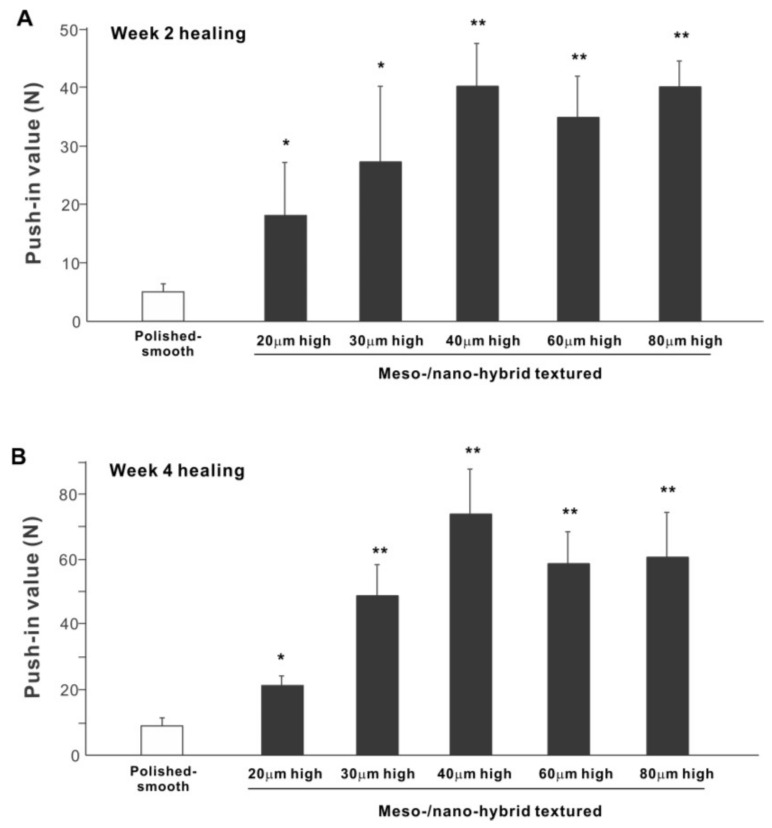
The biomechanical push-in test in the rat femur model at week 2 (**A**) and 4 (**B**) post-implantation. * *p* < 0.05, ** *p* < 0.01, statistically significant difference compared with a polished-smooth surface.

The biomechanical strength of bone–implant integration is the most critical and relevant factor for evaluating the performance of an implant as a load-anchoring device. The strength of bone–implant integration assessed by biomechanical push-in testing at an early stage of healing, two weeks post-implantation, was considerably higher for all meso-/nano-scale hybrid textured implants than the polished-smooth implants (Figure 10A). The push-in values increased with the spike height until the spikes reached 40 μm high and plateaued afterward. The push-in value for implants with 40 μm-high spikes was 8 times greater than for the polished implants. It was also noteworthy that the bone integration of 40 μm-high spiked implants was over twice that of 20 μm-high spiked implants. Similarly, during the later stages of healing, after four weeks, the push-in values for the hybrid-textured implants far exceeded that of the polished implants (Figure 10B). The greatest push-in value was noted for the implants with 40 μm-high spikes, dominating the values of the polished implants and 20 μm-spiked implants by 7 and 3.5 times, respectively.

**Figure 11:**
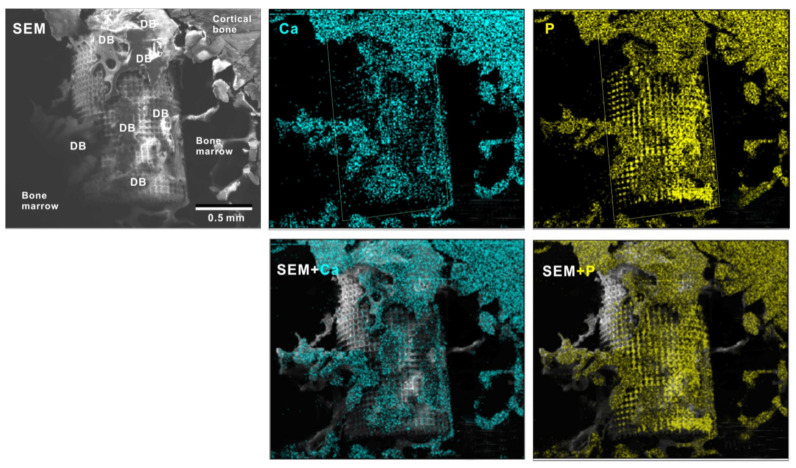
Peri-implant tissue morphology and chemistry around a zirconia implant. To verify bone formation around implants, selected implants were analyzed after the push-in test. A representative result from a meso-/nano-scale hybrid textured implant with 40 μm-high spikes is presented here. SEM images and elemental mapping for Ca and P signals as well as their superimposed images are shown.

To verify the bone formation around zirconia implants, selected implants were examined for tissue morphology and chemistry after push-in testing (Figure 11). The hybrid-textured implants with 40 μm-high spikes were extensively covered with biological structures, as shown in the SEM image. The structures in the top half of the implant originated from the innate cortical bone and/or periosteum and spread to the implant surface, whereas the ones on the bottom half appeared to have stemmed from the implant interface within the bone marrow. The majority of the biological structures were positive for Ca and P signals in the elemental mapping, providing evidence that there was extensive bone formation around the implant.

### 2.6. Technology Validation for Clinical Translation

**Figure 12:**
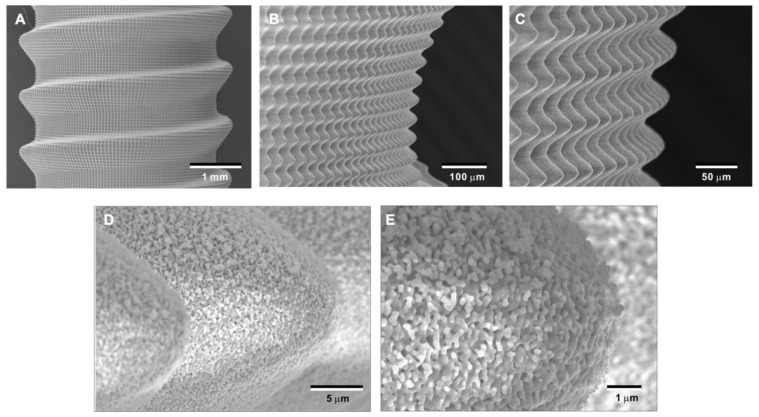
Prototype development of a medical device with the bio-inspired meso-/nano-scale hybrid textured surface for human applications. To verify the viability of the laser technology introduced here in creating medical devices, a zirconia dental implant was developed. A screw-shaped implant, made from Y-TZP, was formed to represent a standardly sized and shaped dental implant with macroscopic helical threads (**A**). After laser etching, the implant vividly presents cactus-inspired meso-spikes (**B**,**C**) and trabecular bone-like nano-scale interwoven architecture (**D**,**E**).

To overcome potential challenges in creating the bio-inspired meso-/nano-scale hybrid textured zirconia surface on future medical devices, a prototype dental implant for human use was developed. The prototype, which was 4 mm in diameter and 10 mm in length, was screw-shaped with macroscopic helical threads to represent a size and shape standard for dental implants. Solid-state laser etching was applied to form 40 μm-high spikes as optimized in the experiments described above. Low-magnification SEM images depicted uniform and seamless formation of cactus-inspired meso-spikes all over the threaded implant surface (Figure 12A–C). There were no visible irregularities or defects in the spikes in any areas (i.e., peak, flank, and valley regions) of the macroscopic threads. The spikes created a controlled, oscillating configuration with a repetitive, peak-and-valley pattern. High-magnification images confirmed the formation of fully networked nano-trabeculae, just as seen on experimental samples, evenly and consistently appearing all over the implant surface (Figure 12D,E).

## 3. Discussion

**Figure 13:**
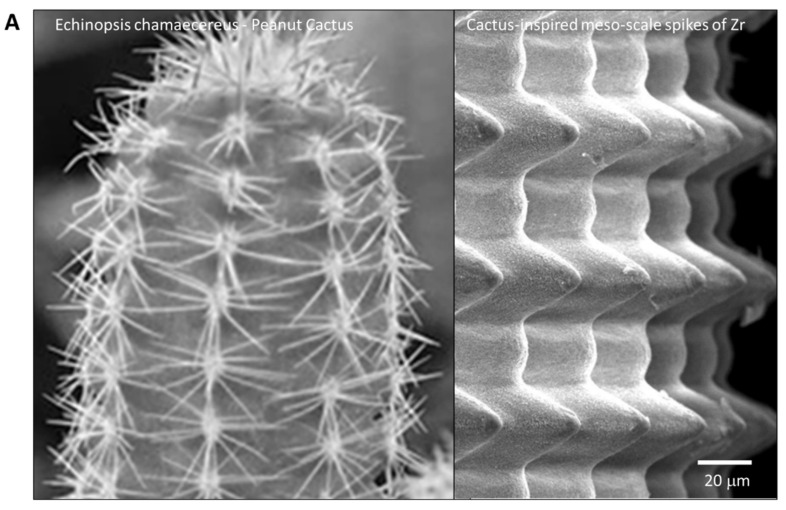

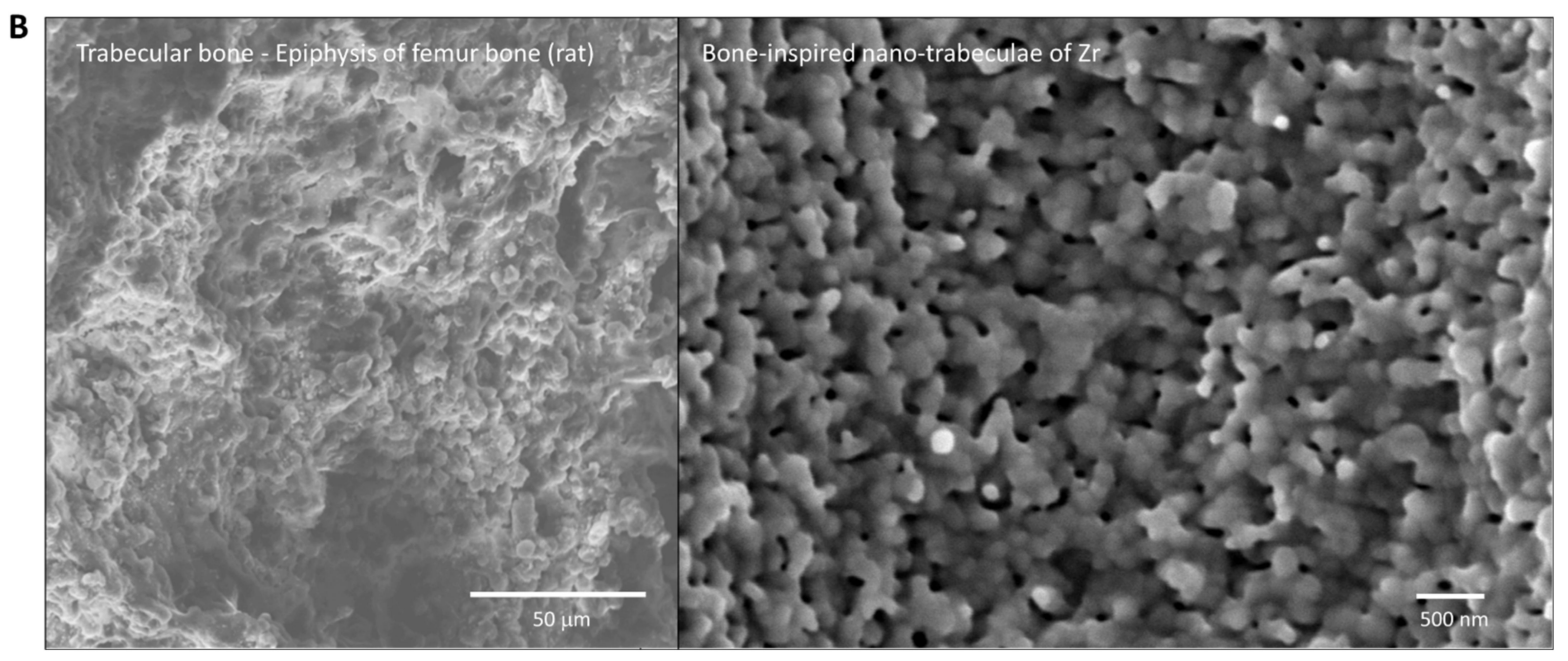
Biomimetic zirconia surface at dual meso- and nano-scale levels. (**A**) Cactus-inspired meso-scale spikes created by solid-state laser etching in the present study. *Echinopsis chamaecereus* peanut cactus (photograph) and the zirconia cylinder with 40 μm-high spikes (SEM image) are presented. (**B**) Bone-inspired nano-scale trabeculae created by solid-state laser etching in the present study. Trabecular bone in the epiphysis of rat femur (SEM image) and the surface of a zirconia cylinder with 40 μm-high spikes (high-magnification SEM image) are presented.

To the best of our knowledge, this is the first study that has created meso- and nano-scale hybrid textured surfaces potentially applicable to implant devices. The meso- and nano-architectures were both biomimetically designed, inspired by cactus and trabecular bone, respectively (Figure 13A,B). The dimensions of the meso-scale spikes were shown to be precisely controllable, which enabled us to optimize the architecture for enhanced bone-implant integration.

In vitro and in vivo results consistently indicated that the surface with 40 μm-high spikes was the best among the design variations tested. Astoundingly, the height of meso-spikes and the performance of the implants, including the strength of the bone-implant integration and the levels of various differentiation markers, were not linearly correlated. In other words, the principle of “the rougher, the better” did not apply to the optimization of the meso-scale texture. We believe this discovery will be a cornerstone of future implant design.

The strength of bone-implant integration achieved with the 40 μm-high spiked zirconia surface deserves considerable attention. The push-in values of implants with this surface texture were approximately 40 N and 72 N after 2 and 4 weeks of healing, respectively. This was a major advancement in surface science and technology for implants, because the push-in values of acid-etched micro-rough titanium, one of the most commonly used surfaces in current dental implants, measured in the same animal model, were reportedly 15 N and 28 N after 2 and 4 weeks of healing, respectively [24,34,35,36]. A primary contributing factor in this breakthrough was conceivably the addition of meso-scale roughness. As mentioned in the Introduction, we devised a strategy of creating meso-scale roughness to enable an implant surface to interlock and engage more with the surrounding bone, which was shown in our testing to be highly effective. Moreover, the surface area of the 40 μm-high spiked surface was increased by 2.63 times relative to a polished-smooth surface, which obviously increased the interfacial area between the implant and bone, and thereby enhanced the strength of bone-implant integration. Even the 30 μm-high spiked zirconia surface outperformed the acid-etched micro-rough titanium by far [24], which re-affirmed the effectiveness and significance of adding the meso-scale spike architecture to enhance bone-to-implant integration, providing a new design strategy for future endosseous implants.

The hybrid-textured surfaces promoted osteoblastic differentiation and minimized the reduction in the number of osteoblasts available for peri-implant bone regeneration, which may explain the enhanced bone-implant integration. All indicators of osteoblastic differentiation, such as ALP activity, matrix calcium deposition, and gene expression, increased with the addition of meso-spikes of any height and peaked when the spikes were 40 μm high, which was convincingly supported the strength of bone-implant integration. The number of osteoblasts is determined by two elements: the number of cells attached to the implant surface and the subsequent rate of their proliferation. According to the literature, there is an inverse correlation between these two elements and surface roughness of implants, i.e., the rougher implant surfaces are, the fewer osteoblasts attach to them, and the rougher implant surfaces are, the lower the rate of osteoblastic proliferation [14,23,30,34,37,38]. This is a disadvantage of rough surfaces over smooth ones, although rough surfaces promote osteoblastic differentiation, as shown in the present results. In fact, the present result was consistent with the principle that lower numbers of cells attached to hybrid-textured surfaces than to a polished surface by day 2. Moreover, the cell density measured on day 5 was lower on the hybrid-rough surfaces. However, the difference between the hybrid-textured surfaces and the polished surface was less than the 3 to 5-fold difference reported between micro-rough and machined-smooth titanium surfaces, where the micro-rough surface showed an average roughness (Sa) of 0.5 to 1.5 μm [30]. This improvement may be ascribed to the increased surface area created by the hybrid texturing counteracting this effect. The incrementally increased surface area from 2.15 to 4.43 times that of a polished surface may have offset the negative impact by increasing the surface roughness from 4.01 to 18.15. For instance, the cell density on day 5 on the hybrid-textured surface with 40 μm-high spikes was only 15% lower than on the polished surface, which is a surprisingly small decrease considering the Sa increased from 0.10 μm to 7.72 μm.

The definitive reasons behind the increased strength of bone and implant integration by the meso- and nano-scale hybrid texturing remain to be explored. Specifically, it is unknown which is the primary contributing factor between the increased mechanical interlocking by the meso-spikes and the increased bone and implant contact. We postulate the plausible contributions of both factors. As mentioned earlier, meso-scale morphology has rarely been implemented in implant devices [20,39], providing no example on its effect. However, considering the macro-morphology, such as implant threads and macro pores, implemented extensively in implant products to increase their overall stability, meso-scale morphology is highly likely to enhance the bone and implant interfacial strength [21,40]. In light of the bone and implant contact, the rate of bone formation around implants is known to be corroborated with the rate of osteoblastic differentiation and the number of osteoblasts [9,22,23,25,26,30,41]. The present in vitro results on the upregulated expression of osteoblastic genes, increased ALP activity and calcium deposition, and increased number of cells suggested the bone formation around the hybrid-textured zirconia surfaces was promoted. We previously established a detailed protocol and analytical parameters for bone morphometry around titanium implants using non-decalcified histological sections [22,25,34,35,42]. However, it is extremely difficult to prepare such ground sections from mini-zirconia implant specimens. Other quantitative methods, such as microCT [32,43,44,45], elemental detection [20,24,27,46,47,48], and biomechanical testing [27,28,29,30,31,43,46,49] based bone morphometry may be considered to localize and quantify the degree of bone formation in future studies.

The cell differentiation on hybrid-textured zirconia was elevated relative to the polished-smooth zirconia thanks to a synergistic effect of the meso- and nano-architectures. It was evident from the remarkable changes in gene expression that the height of the meso-spikes regulated osteoblastic differentiation. However, because creation of the meso-spikes simultaneously creates the nano-trabeculae, it was not possible to determine the effect of nano-trabeculae alone. The levels of collagen 1 and osteocalcin expression at day 15, a relatively late stage, showing remarkable upregulation from the polished surface to the surface with relatively low 20 μm-high spikes suggests that there may be a significant effect of nano-trabeculae. Reports have suggested that different types of nano-textures can promote osteoblastic differentiation differently on titanium, zirconia, and bio-polymer materials [9,11,13,15,20,50,51,52]. The nano-architecture created in the present study is different from previously reported textures and created on a different base material. Therefore, another technological breakthrough enabling the creation of only nano-trabeculae without forming meso-spikes on zirconia is required to address these questions.

In addition, the initial behavior of osteogenic cells on biomaterial surfaces determines the subsequent activity of proliferation and differentiation. Using methods reported previously [14,15,53,54,55,56,57], we attempted cytomorphometry of osteoblasts on the hybrid-textured zirconia surfaces to examine their attaching and spreading behaviors but failed to capture images due to the unprecedented meso-scale vertical profile of the zirconia surfaces. The crisscrossing laser etching created not only a larger surface area but also unique 3-dimensional features, such as an inverted cone depression, wavy surface, and inclines, whose dimensions were equivalent to or larger than the size of osteoblasts. From this perspective, this study presented novel findings on the osteoblastic behavior and response at this unique interface. We hypothesized that such topographical features influenced the attachment and colonization, and subsequent proliferation and differentiation of osteoblasts. Future studies to examine the potential effect of these features on signaling cascades from surface sensing to the cellular spread and adhesion and various other functional phenotypes will of great interest [17,58,59].

Laser etching technology provided an opportunity to design and create a biomimetic meso- and nano-scale hybrid-textured zirconia in this study. As we hypothesized, crisscrossing laser etching was able to engrave cactus prickle-like spikes in a smooth, repetitive, and uniform configuration (Figure 12). Further, the cactus-inspired spikes and surrounding flanks and valleys were covered in their entirety by an unprecedented nano-scale trabecular bone-like architecture where nano-scale random structures resembling nodules, pillars, and villi are tightly interwoven (Figure 12 and Figure 13). This technology may be applicable to different types of medical-grade metallic materials, such as titanium and chromium–cobalt alloy, as well as polymer-based biomaterials, such as poly(D, L-lactic acid) (PLA), poly(D, L-lactic-co-glycolic acid) (PLGA), and polyether ether ketone (PEEK). The technology, design strategy, and results of the biological characterization presented in this study may provide a novel platform for future development of implants and other biomaterial surfaces.

## 4. Materials and Methods

### 4.1. Zirconia Samples and Surface Characterization

Zirconia experimental samples in disk (20 mm diameter, 1.5 mm thickness) and cylindrical form (1 mm diameter, 2 mm length), prepared from yttria-stabilized tetragonal zirconia polycrystal (Y-TZP), were polished and assigned as the “polished-smooth surface” group. To create dual-scale textured surfaces, polished zirconia samples were treated with solid-state laser etching. The laser etching was established to carve grooves with hemispherical bottom surfaces [20]. The laser etching was conducted in a crisscrossing manner to create cactus prickle-like projections, as strategized in Figure 1. The width of the grooves was fixed at 60 μm, while the depth was incrementally varied to make projections of 20, 30, 40, 60, and 80 μm in height. All samples were manufactured and provided by Nantoh Co., Ltd. (Numazu, Japan) and sterilized by autoclaving before cell culture and animal studies. Surface morphology was examined by scanning electron microscopy (SEM; Nova 230 Nano SEM, FEI, Hillsboro, OR, USA) and an optical profile microscope (MeX, Alicona Imaging GmbH, Raaba, Graz, Austria) for three-dimensional imaging, profiling, and quantitative roughness analysis. The average roughness (Sa), peak-to-valley roughness (Sz), and surface area were calculated.

### 4.2. Osteoblast Cell Culture

Bone marrow-derived osteoblasts were isolated from the femurs of 8-week-old male Sprague–Dawley rats and placed into alpha-modified Eagle’s medium supplemented with 15% fetal bovine serum, 50 μg/mL ascorbic acid, 10 mM Na-ß-glycerophosphate, 10^−8^ M dexamethasone, and antibiotic–antimycotic solution containing 10,000 units/mL penicillin G sodium, 10,000 mg/mL streptomycin sulfate, and 25 mg/mL amphotericin B. Cells were incubated in a humidified atmosphere of 95% air and 5% CO_2_ at 37 °C. At 80% confluency, the cells were detached using 0.25% trypsin–1 mM EDTA-4Na and seeded onto zirconia disks placed in 12-well culture dishes at a density of 3 × 10^4^ cells/cm^2^. The culture medium was renewed every three days.

### 4.3. Osteoblast Attachment and Proliferation Assays

The number of osteoblasts attached to the zirconia surfaces during the initial stage of culture was evaluated using a tetrazolium salt (WST-1)-based colorimetric assay (WST-1; Roche Applied Science, Mannheim, Germany) on day 2 of culture as described elsewhere [16,34,45,60]. To evaluate the proliferative activity of osteoblasts, the density of propagated cells was also quantified using a WST-1 assay on day 5.

### 4.4. Alkaline Phosphatase (ALP) Activity

Osteoblast ALP activity was examined on day 10 using two methods of chemical detection: colorimetry and a staining-based assay. As previously described [26,27,61], the cultured cells were rinsed with ddH_2_O and 250 μL of p-nitrophenyl phosphate was added, followed by incubation at 37 °C for 15 min. ALP activity was evaluated by measuring the released nitrophenol in the enzymatic reaction and determined at 405 nm using a plate reader (Biotek). For staining, cultured osteoblasts were washed twice with Hanks’ solution and then incubated with 120 mM Tris buffer (pH 8.4) containing 0.9 mM naphthol AS-MX phosphate and 1.8 mM fast red TR for 30 min at 37 °C.

### 4.5. Matrix Ca Deposition

The mineralization capability of cultured osteoblasts was examined by colorimetry-based quantification of calcium deposition on day 15. The cultures were washed with PBS and incubated overnight in 1 mL of 0.5 mM HCl solution with gentle shaking. The solution was mixed with o-cresolphthalein complexone in an alkaline medium (calcium binding and buffer reagent; Sigma, St. Louis, MO, USA) to produce a red calcium cresolphthalein complexone complex. Color intensity was measured by an ELISA plate reader at 575 nm absorbance.

### 4.6. Real-Time Quantitative Polymerase Chain Reaction (qPCR)

Gene expression was analyzed using qPCR on days 5, 10, and 15. Total RNA was extracted from cells using TRIzol (Invitrogen, Carlsbad, CA, USA) and a Direct-zol RNA MiniPrep kit (Zymo Research, Irvine, CA, USA). Extracted RNA was reverse transcribed into first-strand cDNA using SuperScript III Reverse Transcriptase (Invitrogen). Quantitative PCR was performed in a 20 μL volume containing 90 ng cDNA, 10 μL TaqMan Universal Master Mix II, and 1 μL TaqMan Gene Expression Assay using a QuantStudio 3 Real-Time PCR System (Thermo Fisher Scientific, Canoga Park, CA, USA), to quantify the expression of type I collagen and osteocalcin mRNA. *Gapdh* expression was used as the endogenous control.

### 4.7. Surgery

Eight-week-old male Sprague-Dawley rats were anesthetized with 1–2% isoflurane inhalation. After their legs were shaved and scrubbed with 10% povidone-iodine solution, the distal aspects of the femurs were carefully exposed via skin incision and muscle dissection. The flat surfaces of the distal femurs were selected for implant placement. The implant site was prepared 9 mm from the distal edge of the femur by drilling with a 0.8 mm round burr and enlarged using reamers (#ISO 090 and 100). Profuse irrigation with sterile isotonic saline solution was used for cooling and cleaning. One of the variously textured zirconia implants (1 mm in diameter and 2 mm in length) was placed into a prepared hole. Surgical sites were then closed in layers. Muscle and skin were sutured separately with resorbable suture thread. The University of California at Los Angeles (UCLA) Chancellor’s Animal Research Committee approved this protocol, and all experimentation was performed in accordance with the United States Department of Agriculture (USDA) guidelines on animal research (ARC #2005-175-41E, approved on 30 January 2018).

### 4.8. Implant Biomechanical Push-In Test

The established implant biomechanical push-in test was used to assess the biomechanical strength of bone–implant integration [12,24,25]. At weeks 2 and 4 of healing, femurs containing cylindrical implants were harvested and embedded into autopolymerizing resin with the top surface of the implant parallel to the ground. A testing machine (Instron 5544 electro-mechanical testing system, Instron, Canton, MA, USA) equipped with a 2000 N load cell and a pushing rod (0.8 mm in diameter) was used to load the implant vertically downward at a crosshead speed of 1 mm/min. The push-in value was determined by measuring the peak of the load–displacement curve.

### 4.9. Morphological and Elemental Analyses of Implant/Tissue Complex

After the push-in test, implants were carefully exposed and soaked in agitated water for one hour and dried under heat and vacuum. After being carbon sputter-coated, the specimens were examined by scanning electron microscopy (SEM). The elemental composition of the tissue remnants and the implant interface were analyzed by energy dispersive X-ray spectroscopy (EDX) (UltraDry EDS Detector and Noran System 6, Thermo Fisher Scientific).

### 4.10. Statistical Analyses

Data on surface roughness parameters were collected from six sites on three different disks (*n* = 6). Three disks were used for all cell culture studies (*n* = 3). Six implants were used for the biomechanical push-in test (*n* = 6) for each of the implant surfaces at each healing time point. One-way ANOVA was performed to examine the differences among differently textured surfaces. When appropriate, Bonferroni’s test was used as a post-hoc test. *p*-values less than 0.05 were considered statistically significant.

## 5. Conclusions

We have created a meso-/nano- dual-scale bio-inspired zirconia surface by laser etching. The surface carries cactus-inspired meso-scale spikes and bone-inspired nano-trabeculae. The height of the meso-spikes is controllable and in this study was varied incrementally from 20 to 80 μm. The function of osteoblasts and the bone-implant integration responded in a non-linear fashion to the spike height and peaked or plateaued when the spikes were 40 μm high. These 40 μm-high meso-spikes increased Sa to 7.72 μm from the baseline level of 0.10 μm for a polished-smooth zirconia surface, and likewise increased the surface area by 2.64 times. The dual-scale bio-inspired texturing was proven feasible on the complex surface of a prototype dental implant, providing a novel, biomimetic platform technology and design strategy for developing dental and orthopedic implants and other biomaterials.

## Data Availability

Data availability on request from author.

## References

[1] Shao F., Wu Y., Tian Z., Liu S. (2021). Biomimetic nanoreactor for targeted cancer starvation therapy and cascade amplificated chemotherapy. Biomaterials.

[2] Wang X., Gao B., Chan B.P. (2021). Multiphoton microfabrication and micropatterning (MMM)—An all-in-one platform for engineering biomimetic soluble cell niches. Biomaterials.

[3] Bai J., Wang H., Chen H., Ge G., Wang M., Gao A., Tong L., Xu Y., Yang H., Pan G. (2020). Biomimetic osteogenic peptide with mussel adhesion and osteoimmunomodulatory functions to ameliorate interfacial osseointegration under chronic inflammation. Biomaterials.

[4] Zhu Y., Kong L., Farhadi F., Xia W., Chang J., He Y., Li H. (2019). An injectable continuous stratified structurally and functionally biomimetic construct for enhancing osteochondral regeneration. Biomaterials.

[5] Wang T., Zhai Y., Nuzzo M., Yang X., Yang Y., Zhang X. (2018). Layer-by-layer nanofiber-enabled engineering of biomimetic periosteum for bone repair and reconstruction. Biomaterials.

[6] Zhou X., Sahai N., Qi L., Mankoci S., Zhao W. (2015). Biomimetic and nanostructured hybrid bioactive glass. Biomaterials.

[7] Fritschen A., Blaeser A. (2021). Biosynthetic, biomimetic, and self-assembled vascularized Organ-on-a-Chip systems. Biomaterials.

[8] Holzwarth J.M., Ma P.X. (2011). Biomimetic nanofibrous scaffolds for bone tissue engineering. Biomaterials.

[9] Yamada M., Ueno T., Tsukimura N., Ikeda T., Nakagawa K., Hori N., Suzuki T., Ogawa T. (2012). Bone integration capability of nanopolymorphic crystalline hydroxyapatite coated on titanium implants. Int. J. Nanomed..

[10] Tsukimura N., Ueno T., Iwasa F., Minamikawa H., Sugita Y., Ishizaki K., Ikeda T., Nakagawa K., Yamada M., Ogawa T. (2011). Bone integration capability of alkali- and heat-treated nanobimorphic Ti–15Mo–5Zr–3Al. Acta Biomater..

[11] Tsukimura N., Yamada M., Iwasa F., Minamikawa H., Att W., Ueno T., Saruwatari L., Aita H., Chiou W.-A., Ogawa T. (2011). Synergistic effects of UV photofunctionalization and micro-nano hybrid topography on the biological properties of titanium. Biomaterials.

[12] Ueno T., Tsukimura N., Yamada M., Ogawa T. (2011). Enhanced bone-integration capability of alkali- and heat-treated nanopolymorphic titanium in micro-to-nanoscale hierarchy. Biomaterials.

[13] Kubo K., Tsukimura N., Iwasa F., Ueno T., Saruwatari L., Aita H., Chiou W.-A., Ogawa T. (2009). Cellular behavior on TiO_2_ nanonodular structures in a micro-to-nanoscale hierarchy model. Biomaterials.

[14] Hori N., Iwasa F., Ueno T., Takeuchi K., Tsukimura N., Yamada M., Hattori M., Yamamoto A., Ogawa T. (2010). Selective cell affinity of biomimetic micro-nano-hybrid structured TiO_2_ overcomes the biological dilemma of osteoblasts. Dent. Mater..

[15] Hori N., Iwasa F., Tsukimura N., Sugita Y., Ueno T., Kojima N., Ogawa T. (2011). Effects of UV photofunctionalization on the nanotopography enhanced initial bioactivity of titanium. Acta Biomater..

[16] Iwasa F., Tsukimura N., Sugita Y., Kanuru R.K., Kubo K., Hasnain H., Att W., Ogawa T. (2011). TiO2 micro-nano-hybrid surface to alleviate biological aging of UV-photofunctionalized titanium. Int. J. Nanomed..

[17] Miyauchi T., Yamada M., Yamamoto A., Iwasa F., Suzawa T., Kamijo R., Baba K., Ogawa T. (2010). The enhanced characteristics of osteoblast adhesion to photofunctionalized nanoscale TiO_2_ layers on biomaterials surfaces. Biomaterials.

[18] Ogawa T., Saruwatari L., Takeuchi K., Aita H., Ohno N. (2008). Ti Nano-nodular Structuring for Bone Integration and Regeneration. J. Dent. Res..

[19] Sugita Y., Ishizaki K., Iwasa F., Ueno T., Minamikawa H., Yamada M., Suzuki T., Ogawa T. (2011). Effects of pico-to-nanometer-thin TiO_2_ coating on the biological properties of microroughened titanium. Biomaterials.

[20] Rezaei N.M., Hasegawa M., Ishijima M., Nakhaei K., Okubo T., Taniyama T., Ghassemi A., Tahsili T., Park W., Hirota M. (2018). Biological and osseointegration capabilities of hierarchically (meso-/micro-/nano-scale) roughened zirconia. Int. J. Nanomed..

[21] Att W., Tsukimura N., Suzuki T., Ogawa T. (2007). Effect of supramicron roughness characteristics produced by 1- and 2-step acid etching on the osseointegration capability of titanium. Int. J. Oral Maxillofac. Implant..

[22] Ogawa T., Nishimura I. (2003). Different bone integration profiles of turned and acid-etched implants associated with modulated expression of extracellular matrix genes. Int. J. Oral Maxillofac. Implant..

[23] Ogawa T., Nishimura I. (2006). Genes Differentially Expressed in Titanium Implant Healing. J. Dent. Res..

[24] Ogawa T., Ozawa S., Shih J.-H., Ryu K., Sukotjo C., Yang J.-M., Nishimura I. (2000). Biomechanical Evaluation of Osseous Implants Having Different Surface Topographies in Rats. J. Dent. Res..

[25] Ozawa S., Ogawa T., Iida K., Sukotjo C., Hasegawa H., Nishimura R., Nishimura I. (2002). Ovariectomy hinders the early stage of bone-implant integration: Histomorphometric, biomechanical, and molecular analyses. Bone.

[26] Tsukimura N., Kojima N., Kubo K., Att W., Takeuchi K., Kameyama Y., Maeda H., Ogawa T. (2008). The effect of superficial chemistry of titanium on osteoblastic function. J. Biomed. Mater. Res. Part A.

[27] Nakamura H., Saruwatari L., Aita H., Takeuchi K., Ogawa T. (2005). Molecular and Biomechanical Characterization of Mineralized Tissue by Dental Pulp Cells on Titanium. J. Dent. Res..

[28] Nakamura H., Shim J., Butz F., Aita H., Gupta V., Ogawa T. (2006). Glycosaminoglycan degradation reduces mineralized tissue–titanium interfacial strength. J. Biomed. Mater. Res. Part A.

[29] Nakamura H., Butz F., Saruwatari L., Ogawa T. (2007). A role for proteoglycans in mineralized tissue-titanium adhesion. J. Dent. Res..

[30] Takeuchi K., Saruwatari L., Nakamura H.K., Yang J.-M., Ogawa T. (2005). Enhanced intrinsic biomechanical properties of osteoblastic mineralized tissue on roughened titanium surface. J. Biomed. Mater. Res. Part A.

[31] Butz F., Aita H., Wang C., Ogawa T. (2006). Harder and Stiffer Bone Osseointegrated to Roughened Titanium. J. Dent. Res..

[32] Butz F., Ogawa T., Chang T.-L., Nishimura I. (2006). Three-dimensional bone-implant integration profiling using micro-computed tomography. Int. J. Oral Maxillofac. Implant..

[33] Att W., Takeuchi M., Suzuki T., Kubo K., Anpo M., Ogawa T. (2009). Enhanced osteoblast function on ultraviolet light-treated zirconia. Biomaterials.

[34] Aita H., Hori N., Takeuchi M., Suzuki T., Yamada M., Anpo M., Ogawa T. (2009). The effect of ultraviolet functionalization of titanium on integration with bone. Biomaterials.

[35] Att W., Hori N., Takeuchi M., Ouyang J., Yang Y., Anpo M., Ogawa T. (2009). Time-dependent degradation of titanium osteoconductivity: An implication of biological aging of implant materials. Biomaterials.

[36] Ueno T., Yamada M., Hori N., Suzuki T., Ogawa T. (2010). Effect of ultraviolet photoactivation of titanium on osseointegration in a rat model. Int. J. Oral Maxillofac. Implant..

[37] Att W., Ogawa T. (2012). Biological aging of implant surfaces and their restoration with ultraviolet light treatment: A novel understanding of osseointegration. Int. J. Oral Maxillofac. Implant..

[38] Saruta J., Sato N., Ishijima M., Okubo T., Hirota M., Ogawa T. (2019). Disproportionate Effect of Sub-Micron Topography on Osteoconductive Capability of Titanium. Int. J. Mol. Sci..

[39] Hasegawa M., Saruta J., Hirota M., Taniyama T., Sugita Y., Kubo K., Ishijima M., Ikeda T., Maeda H., Ogawa T. (2020). A Newly Created Meso-, Micro-, and Nano-Scale Rough Titanium Surface Promotes Bone-Implant Integration. Int. J. Mol. Sci..

[40] Uno M., Hayashi M., Ozawa R., Saruta J., Ishigami H., Ogawa T. (2020). Mechanical Interlocking Capacity of Titanium with Respect to Surface Morphology and Topographical Parameters. J. Dent. Oral Biol..

[41] Ogawa T. (2012). UV-photofunctionalization of titanium implants. Oral Craniofacial Tissue Eng..

[42] Sugita Y., Okubo T., Saita M., Ishijima M., Torii Y., Tanaka M., Iwasaki C., Sekiya T., Tabuchi M., Mohammadzadeh Rezaei N. (2020). Novel Osteogenic Behaviors around Hydrophilic and Radical-Free 4-META/MMA-TBB: Implications of an Osseointegrating Bone Cement. Int. J. Mol. Sci..

[43] Butz F., Ogawa T., Nishimura I. (2011). Interfacial shear strength of endosseous implants. Int. J. Oral Maxillofac. Implant..

[44] Nishimura I., Huang Y., Butz F., Ogawa T., Lin A., Wang C.J. (2007). Discrete deposition of hydroxyapatite nanoparticles on a titanium implant with predisposing substrate microtopography accelerated osseointegration. Nanotechnology.

[45] Ueno T., Yamada M., Suzuki T., Minamikawa H., Sato N., Hori N., Takeuchi K., Hattori M., Ogawa T. (2010). Enhancement of bone–titanium integration profile with UV-photofunctionalized titanium in a gap healing model. Biomaterials.

[46] Saruwatari L., Aita H., Butz F., Nakamura H.K., Ouyang J., Yang Y., Chiou W.-A., Ogawa T. (2005). Osteoblasts Generate Harder, Stiffer, and More Delamination-Resistant Mineralized Tissue on Titanium Than on Polystyrene, Associated With Distinct Tissue Micro- and Ultrastructure. J. Bone Miner. Res..

[47] Taniyama T., Saruta J., Rezaei N.M., Nakhaei K., Ghassemi A., Hirota M., Okubo T., Ikeda T., Sugita Y., Hasegawa M. (2020). UV-Photofunctionalization of Titanium Promotes Mechanical Anchorage in A Rat Osteoporosis Model. Int. J. Mol. Sci..

[48] Suzuki T., Hori N., Att W., Kubo K., Iwasa F., Ueno T., Maeda H., Ogawa T. (2009). Ultraviolet Treatment Overcomes Time-Related Degrading Bioactivity of Titanium. Tissue Eng. Part A.

[49] Butz F., Aita H., Takeuchi K., Ogawa T. (2005). Enhanced mineralized tissue adhesion to titanium over polystyrene assessed by the nano-scratch test. J. Biomed. Mater. Res. Part A.

[50] Dalby M.J., Gadegaard N., Tare R., Andar A., Riehle M., Herzyk P., Wilkinson C.D.W., Oreffo R. (2007). The control of human mesenchymal cell differentiation using nanoscale symmetry and disorder. Nat. Mater..

[51] Ikeda T., Hagiwara Y., Hirota M., Tabuchi M., Yamada M., Sugita Y., Ogawa T. (2014). Effect of photofunctionalization on fluoride-treated nanofeatured titanium. J. Biomater. Appl..

[52] Dalby M.J., McCloy D., Robertson M., Agheli H., Sutherland D., Affrossman S., Oreffo R. (2006). Osteoprogenitor response to semi-ordered and random nanotopographies. Biomaterials.

[53] Saruta J., Ozawa R., Hamajima K., Saita M., Sato N., Ishijima M., Kitajima H., Ogawa T. (2021). Prolonged Post-Polymerization Biocompatibility of Polymethylmethacrylate-Tri-n-Butylborane (PMMA-TBB) Bone Cement. Materials.

[54] Yamada M., Kojima N., Paranjpe A., Att W., Aita H., Jewett A., Ogawa T. (2008). N-acetyl Cysteine (NAC)-assisted Detoxification of PMMA Resin. J. Dent. Res..

[55] Ueno T., Takeuchi M., Hori N., Iwasa F., Minamikawa H., Igarashi Y., Anpo M., Ogawa T. (2012). Gamma ray treatment enhances bioactivity and osseointegration capability of titanium, Journal of biomedical materials research. Part B Appl. Biomater..

[56] Ueno T., Ikeda T., Tsukimura N., Ishijima M., Minamikawa H., Sugita Y., Yamada M., Wakabayashi N., Ogawa T. (2016). Novel antioxidant capability of titanium induced by UV light treatment. Biomaterials.

[57] Yamada M., Miyauchi T., Yamamoto A., Iwasa F., Takeuchi M., Anpo M., Sakurai K., Baba K., Ogawa T. (2010). Enhancement of adhesion strength and cellular stiffness of osteoblasts on mirror-polished titanium surface by UV-photofunctionalization. Acta Biomater..

[58] Iwasa F., Baba K., Ogawa T. (2016). Enhanced intracellular signaling pathway in osteoblasts on ultraviolet lighttreated hydrophilic titanium. Biomed. Res..

[59] Iwasa F., Hori N., Ueno T., Minamikawa H., Yamada M., Ogawa T. (2010). Enhancement of osteoblast adhesion to UV-photofunctionalized titanium via an electrostatic mechanism. Biomaterials.

[60] Hamajima K., Ozawa R., Saruta J., Saita M., Kitajima H., Taleghani S.R., Usami D., Goharian D., Uno M., Miyazawa K. (2020). The Effect of TBB, as an Initiator, on the Biological Compatibility of PMMA/MMA Bone Cement. Int. J. Mol. Sci..

[61] Ueno T., Yamada M., Igarashi Y., Ogawa T. (2011). N-acetyl cysteine protects osteoblastic function from oxidative stress. J. Biomed. Mater. Res. Part A.

